# Effect of Soil and Root Extracts on the Innate Immune Response of American Ginseng (*Panax quinquefolius*) to Root Rot Caused by *Ilyonectria mors-panacis*

**DOI:** 10.3390/plants12132540

**Published:** 2023-07-04

**Authors:** Behrang Behdarvandi, Paul H. Goodwin

**Affiliations:** School of Environmental Sciences, University of Guelph, 50 Stone Road East, Guelph, ON N1G 2W, Canada; bbehdarv@uoguelph.ca

**Keywords:** gene expression, ginseng replant disease, immunosuppression, necrotrophy

## Abstract

*Panax quinquefolius* shows much higher mortality to *Ilyonectria mors-panacis* root rot when grown in soil previously planted with ginseng than in soil not previously planted with ginseng, which is known as ginseng replant disease. Treatment of ginseng roots with methanol extracts of previous ginseng soils significantly increased root lesion sizes due to *I. mors-panacis* compared to roots treated with water or methanol extracts of ginseng roots or non-ginseng soils. Inoculation of water-treated roots with *I. mors-panacis* increased expression of a basic chitinase 1 gene (*PqChi-1*), neutral pathogenesis-related protein 5 gene (*PqPR5*) and pathogenesis-related protein 10-2 gene (*PqPR10-2*), which are related to jasmonic acid (JA), ethylene (ET) or necrotrophic infection, and also increased expression of an acidic β-1-3-glucanase gene (*PqGlu*), which is related to salicylic acid (SA). Infection did not affect expression of a cysteine protease inhibitor gene (*PqCPI*). Following infection, roots treated with ginseng root extract mostly showed similar expression patterns as roots treated with water, but roots treated with previous ginseng soil extract showed reduced expression of *PqChi-1*, *PqPR5*, *PqPR10-2* and *PqCPI*, but increased expression of *PqGlu*. Methanol-soluble compound(s) in soil previously planted with ginseng are able to increase root lesion size, suppress JA/ET-related gene expression and trigger SA-related gene expression in ginseng roots during *I. mors-panacis* infection, and may be a factor contributing to ginseng replant disease.

## 1. Introduction

Cultivation of ginseng plants in a field previously used for ginseng production is usually associated with partial reduction or up to 100% loss of the crop, known as ginseng replant disorder or replant disease [1]. It is primarily associated with root rot, which is most commonly due to infection by *Cylindrocarpon destructans* or *C. destructans* f. sp. *panacis* [2]. Based on sequences of six genes commonly used for fungal taxonomy, Cabral et al. [3] created the *I. radicicola* complex, reclassifying the less aggressive ginseng pathogen, *C. destructans,* into *I. crassa*, *I. panacis* and *I. robusta*. The more aggressive ginseng pathogen, *C. destructans* f. sp. *panics* [4], was reclassified as *Ilyonectria mors-panacis*.

Infection of *I. mors-panacis* can cause limited reddish to dark brown root lesions, which can develop into disappearing root rot, where the entire root centre, including the vascular tissues, is rotted, with only the peridermal shell remaining, resulting in reddish wilted stems and leaves and eventually plant death [5]. However, it is unknown why the same pathogen causes much greater levels of plant death in soils previously planted with ginseng. It has been proposed that young ginseng plants in previous ginseng soil are affected by chemicals released into the soil by the roots of the first ginseng crop. For example, Bi et al. [6] found nine phenolic compounds in *P. quinquefolius* soil, such as vanillin, syringic acid and coumaric acid, that inhibited radicle and shoot growth of ginseng seedlings. Xiang et al. [7] reported 44 compounds in consecutively cultivated *P. notoginseng* soils and identified the phenols, benzoic acid and phthalic acid, and the saturated fatty acids, palmitic acid and stearic acid, which significantly inhibited the growth of *P. notoginseng* seedlings. Yang et al. [8] reported that ginsenosides, extracted from ginseng root and ginseng cultivated soil, significantly inhibited the seedling emergence and growth of *P. ginseng*. Ginsenosides extracted from *P. ginseng* roots also inhibited the growth of *P. quinquefolius* seedlings [9]. Thus, it appears that secreted ginsenosides and other compounds are biological active molecules that have negative effects on ginseng roots. However, this hypothesis does not account for the common association of disappearing root rot caused by *I. mors-panacis* with replant disease.

Another possibility is that replant disease is due to ginseng roots growing in previous ginseng soil being less resistant to *I. mors-panacis* than roots in non-ginseng soil. In plants, resistance responses to pathogens can be due to pathogen-associated molecular pattern (PAMP)-triggered immunity (PTI) or effector-triggered immunity (ETI) [10]. PTI and ETI responses are regulated by plant defense hormones, such as jasmonic acid (JA) and ethylene (ET) related to response to necrotrophs and salicylic acid (SA) related to responses to biotrophs [11]. Resistance to root rot diseases is mostly linked to JA and ET, and plants lacking a JA response show decreased resistance, whereas SA has not been linked to resistance to root rots [12]. The expression of certain genes, such as basic PR protein genes, are often used as markers for JA-regulated responses, whereas the expression of acidic PR protein genes are often used as markers for SA-regulated responses. For example, basic chitinase gene (*PgChi*) expression in *P. ginseng* significantly increased when *P. ginseng* roots were treated with JA [13], whereas acidic glucanase gene (*PgGlu-1*) expression was induced by SA [14]. Infection of *P. ginseng* roots with *I. mors-panacis* or *I. robusta* showed that the expression of lipoxygenase 6 (*PgLOX6*) used as a marker for JA increased slightly at 4 dpi with *I. mors-panacis* but greatly with *I. robusta* at 16 dpi [15]. However, the expression of phenylalanine ammonia-lyase 1 (*PgPAL1*) used as a marker for SA increased greatly at 4 and 16 dpi with *I. mors-panacis* but not with *I. robusta* infection. Thus, it appears that *Ilyonectia* infection can affect both JA- and SA-regulated gene expression in ginseng roots.

The goal of this work was to determine if there were compounds in soil previously planted with ginseng that could decrease the resistance of *P. quinquefolius* to root rot caused by *I. mors-panacis* and thus be at least partially responsible for ginseng replant disease. Lesion sizes were compared between roots treated with extracts from soil previously planted with ginseng, roots treated with extracts from soil not previously planted with ginseng, roots treated with extracts from ginseng roots, and roots treated with water. In addition, the expression of several defence genes was examined before and after *I. mors-panacis* infection to determine if the extracts affected defence responses compared to water. The defense genes examined were previously shown to be induced in *P. ginseng* after infection by *Botrytis cinerea*, *Colletotrichum gloeosporioides* or *Phytophthora capsici*, or induced by salt stress, JA or SA [13,16,17,18,19].

## 2. Results

### 2.1. Effects of the Extracts on Root Rot Disease

**Figure 1:**
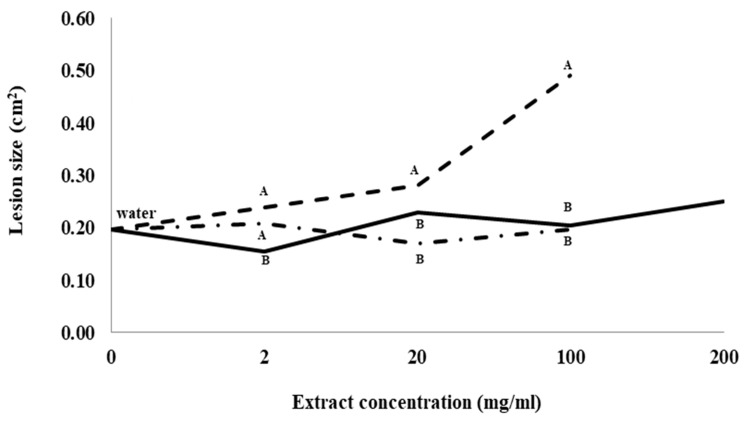
Lesion size of *Ilyonectria mors-panacis* isolate IMP.ND4Z15 on *P. quinquefolius* roots after treatment with water (0 mg/mL), previous ginseng soil extract (**- - - - **), hopyard soil extract (**-·-·-·-**) or ginseng root extract (⸺⸺). Surface sterilized wounded roots of *P. quinquefolius* were treated with 15 µL onto the wound roots for 2 h followed by 15 µL 10^6^ conidia/mL *I. mors-panacis* in water and kept for 12 days. Roots were incubated for 12 dpi at 22 °C in the dark. The lesion sizes were measured by tracing lesions on acetate sheets followed by quantifying using ImageJ software. Means at each extract concentration with the same letter are not significantly different according to Fisher’s LSD at *p* = 0.05. Each data point represents the mean of three replications, with six lesions measured per replication.

Root lesion size caused by *I. mors-panacis* IMP.ND4Z15 was not significantly different between control roots treated with water and any of the concentrations of ginseng root extract or hopyard (soil not previously planted with ginseng) soil extract (Figure 1). Lesion size for roots treated with 2 mg/mL previous ginseng soil extract was significantly higher than roots treated with 2 mg/mL root extract, but not significantly higher than roots treated with 2 mg/mL hopyard soil extract. Lesion size was significantly higher in roots treated with 20 and 100 mg/mL previous ginseng soil extract than in roots treated with either 20 and 100 mg/mL hopyard soil or root extract. Lesion size was more than double (0.49 cm^2^ vs. 0.20 cm^2^) at 100 mg/mL with previous ginseng soil extract compared to both 100 mg/mL root extract or hopyard soil extract treatments. No lesions were observed when the roots were treated with dsH_2_O as a negative control.

### 2.2. Composition of Extracts

No ginsenosides were detected in water or hopyard soil extract. The lack of ginsenosides in hopyard soil is consistent with no ginseng crop being grown previously in that soil. Root extract had seven PPDs and three PPTs detectable, while previous ginseng soil extract had four PPDs and two PPTs detectable with undetectable levels of the PPDs, Rb1, Rc and Rb2, and the PPT, R1 (Table 1). No ginsenosides were detected in previous ginseng soil extract that were not also present in ginseng root extract. All ginsenoside types were significantly lower in previous ginseng soil than in roots, except for GXVII, which was not significantly different.

### 2.3. Effects of Extracts on Gene Expression

**Figure 2:**
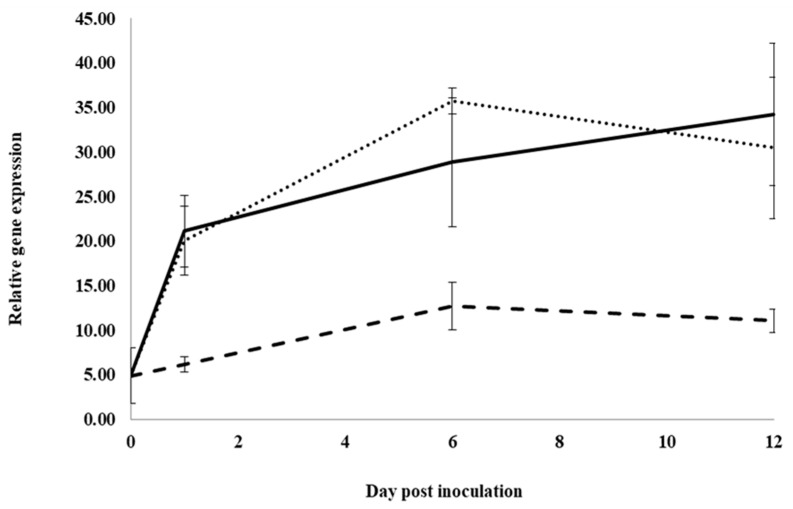
Effect of water, root extract or previous ginseng soil extract on expression of the *P. quinquefolius* basic chitinase 1 gene (*PqChi-1*) in roots following infection by *I. mors-panacis*. Relative RT-PCR of *PqChi1* expression in roots of *P. quinquefolius* treated with water (**······**), 100 mg/mL ginseng root extract (**⸺⸺**) or 100 mg/mL previous ginseng soil extract (**- - - - **), followed 2 h later by inoculation of *I. mors-panacis* isolate IMP.ND4Z15 on the wounded roots. Semiquantitative RT-PCR was performed with co-amplification of *PqChi-1* and a constitutive control, *PqIF3G1*. Each data point represents the mean of three replications, with error bars showing standard deviations.

Control roots treated with water and inoculated with *I. mors-panacis* IMP.ND4Z15 showed a significant increase in the expression of the basic chitinase I gene, *PqChi-1*, from 0 to 1 dpi and 1 to 6 dpi, but there was no significant change from 6 to 12 dpi (Figure 2). At 1, 6 and 12 dpi, expression was not significantly different between inoculated roots treated with water or root extract, whereas expression was significantly lower with inoculated roots treated with previous ginseng soil extract by 3.3-fold at 1 dpi, 2.8- to 2.2-fold at 6 dpi and 2.7- to 3.1-fold at 12 dpi compared to water or root extract. Thus, previous ginseng soil extract appeared to suppress the induced expression of this gene following infection at 1 to 12 dpi.

**Figure 3:**
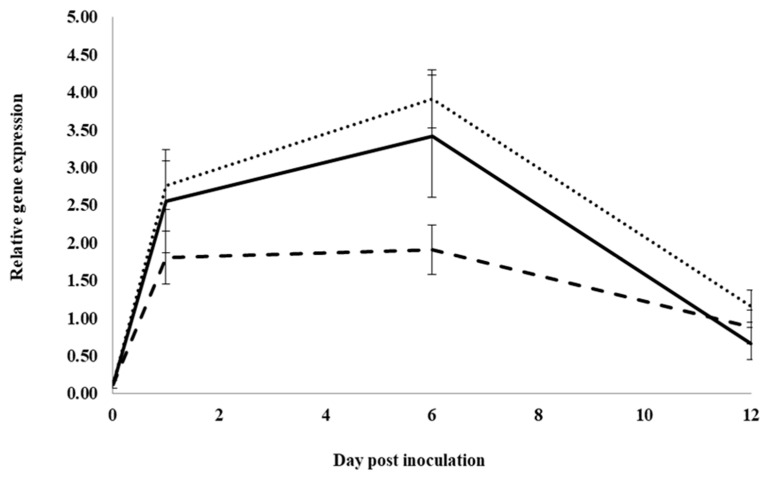
Effect of water, root extract or previous ginseng soil extract on the expression of the *P. quinquefolius* neutral pathogenesis-related protein 5 gene (*PqPR5*) in roots following infection by *I. mors-panacis*. Relative RT-PCR of *PqPR5* expression in roots of *P. quinquefolius* treated with water (**······**), 100 mg/mL ginseng root extract (**⸺⸺**) or 100 mg/mL previous ginseng soil extract (**- - - - **), followed 2 h later by inoculation of *I. mors-panacis* isolate IMP.ND4Z15 on the wounded roots. Semiquantitative RT-PCR was performed with co-amplification of *PqPR5* and a constitutive control, *PqIF3G1*. Each data point represents the mean of three replications, with error bars showing standard deviations.

In control water-treated roots, expression significantly increased for the neutral pathogenesis-related protein 5 gene, *PqnPR5*, from 0 to 1 dpi, but it was not significantly changed between 1 and 6 dpi. It then significantly decreased from 6 to 12 dpi, approaching pre-inoculation levels (Figure 3). At 1, 6 and 12 dpi, expression was not significantly different between water and root extract treatment. However, at 6 dpi, expression in inoculated roots treated with previous ginseng soil extract was significantly lower than in roots treated with water or root extract by 2.05- and 1.8-fold, respectively. Similar to *PqChi-1* expression, previous ginseng soil extract decreased the induction of the expression of *PqnPR5* but only at 6 dpi.

**Figure 4:**
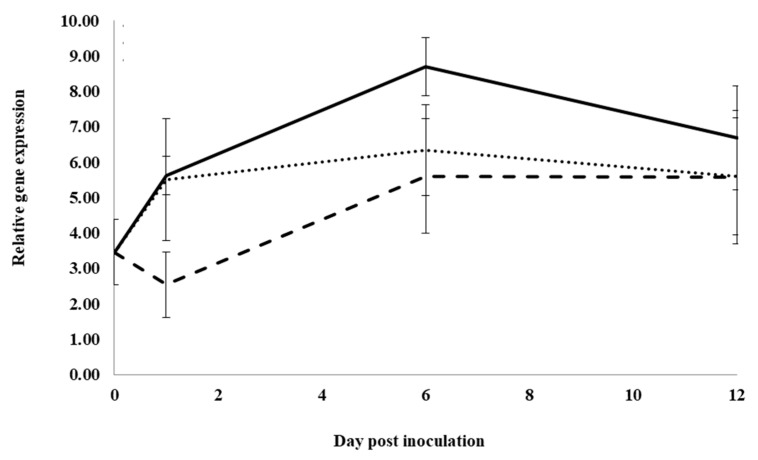
Effect of water, root extract or previous ginseng soil extract on the expression of the *P. quinquefolius* pathogenesis-related protein 10-2 gene (*PqPR10-2*) in roots following infection by *I. mors-panacis*. Relative RT-PCR of *PqPR10-2* expression in roots of *P. quinquefolius* treated with water (**······**), 100 mg/mL ginseng root extract (**⸺⸺**) or 100 mg/mL previous ginseng soil extract (**- - - - **), followed 2 h later by inoculation of *I. mors-panacis* isolate IMP.ND4Z15 on the wounded roots. Semiquantitative RT-PCR was performed with co-amplification of *PqPR10-2* and a constitutive control, *PqIF3G1*. Each data point represents the mean of three replications, with error bars showing standard deviations.

The expression of the pathogenesis-related protein 10 gene, *PqPR10-2*, significantly increased with infection from 0 to 1 dpi, but it did not increase significantly from 1 to 6 dpi and from 6 to 12 dpi in control water-treated roots (Figure 4). At 1 dpi, expression with previous ginseng soil extract was significantly 2.1-fold lower than with water or root extract treatment, which were not significantly different from each other. At 6 dpi, expression was significantly lower in roots treated with water and previous ginseng soil extract, which were not significantly different from each other compared to inoculated roots treated with root extract. At 12 dpi, no significant differences were observed with treatments. Similar to *PqChi-1* and *PqnPR5*, the expression of *PqPR10-2* was induced by infection, but this was suppressed in roots treated with previous ginseng soil extract at 1 dpi.

**Figure 5:**
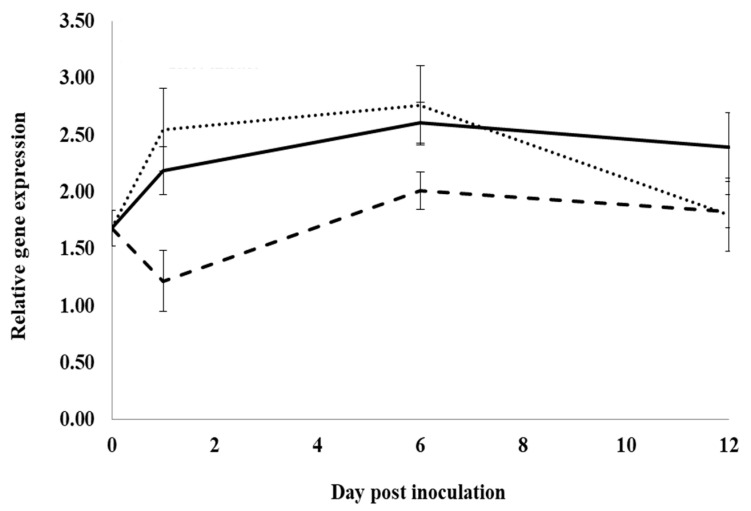
Effect of water, root extract or previous ginseng soil extract on the expression of the *P. quinquefolius* spermidine synthase gene (*PqSPD*) in roots following infection by *I. mors-panacis*. Relative RT-PCR of *PqSPD* expression in roots of *P. quinquefolius* treated with water (**······**), 100 mg/mL ginseng root extract (**⸺⸺**) or 100 mg/mL previous ginseng soil extract (**- - - - **), followed 2 h later by inoculation of *I. mors-panacis* isolate IMP.ND4Z15 on the wounded roots. Semiquantitative RT-PCR was performed with co-amplification of *PqSPD* and a constitutive control, *PqIF3G1*. Each data point represents the mean of three replications, with error bars showing standard deviations.

Infection of control water-treated roots resulted in significantly increased expression of spermidine synthase, *PqSPD*, from 0 to 1dpi, but not from 1 to 6 dpi, and then significantly decreased expression from 6 to 12 dpi, returning to pre-infection levels (Figure 5). At 1 dpi, expression in roots treated with previous ginseng soil extract was significantly lower by 2.1- and 1.8-fold, respectively, compared to expression in roots treated with water or root extract, which were not significantly different from each other. Similarly, at 6 dpi, expression in roots treated with previous ginseng soil extract was significantly lower by 0.7-fold compared to water or root extract treatments, which were not significantly different from each other. At 12 dpi, expression was not significantly different between roots treated with water and previous ginseng soil extract, but expression was significantly higher with root extract compared to water but not with previous ginseng soil extract. Similar to *PqChi-1*, *PqnPR5* and *PqPR10-2*, *PqSPD* expression was induced by infection, but this was suppressed in roots treated with previous ginseng soil extract at 1 and 6 dpi.

**Figure 6:**
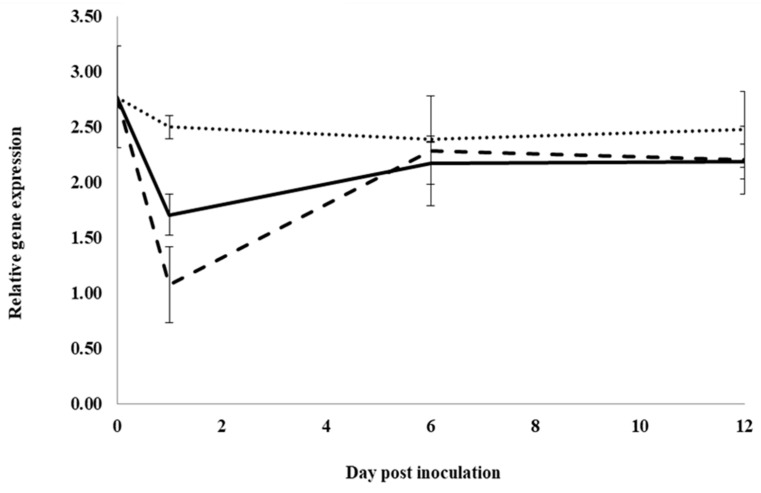
Effect of water, root extract or previous ginseng soil extract on the expression of the *P. quinquefolius* cysteine protease inhibitor gene (*PqCPI*) in roots following infection by *I. mors-panacis*. Relative RT-PCR of *PqCPI* expression in roots of *P. quinquefolius* treated with water (**······**), 100 mg/mL ginseng root extract (**⸺⸺**) or 100 mg/mL previous ginseng soil extract (**- - - - **), followed 2 h later by inoculation of *I. mors-panacis* isolate IMP.ND4Z15 on the wounded roots. Semiquantitative RT-PCR was performed with co-amplification of *PqCPI* and a constitutive control, *PqIF3G1*. Each data point represents the mean of three replications, with error bars showing standard deviations.

Expression of the cysteine protease inhibitor gene, *PqCPI*, was not significantly changed following inoculation for control water-treated roots (Figure 6). At 1 dpi, expression was significantly lower in roots treated with previous ginseng soil extract compared to roots treated with water or root extract by 2.3- and 1.6-fold, respectively, and expression in roots treated with root extract was significantly lower than roots treated with water by 1.5-fold. At 6 and 12 dpi, expression was not significantly different between the treatments. Unlike the other genes, expression of *PqCPI* was not induced by infection at any time point in control roots, but it was still suppressed with previous ginseng soil extract at 1 dpi, although this also occurred to a lesser extent with root extract treatment.

**Figure 7:**
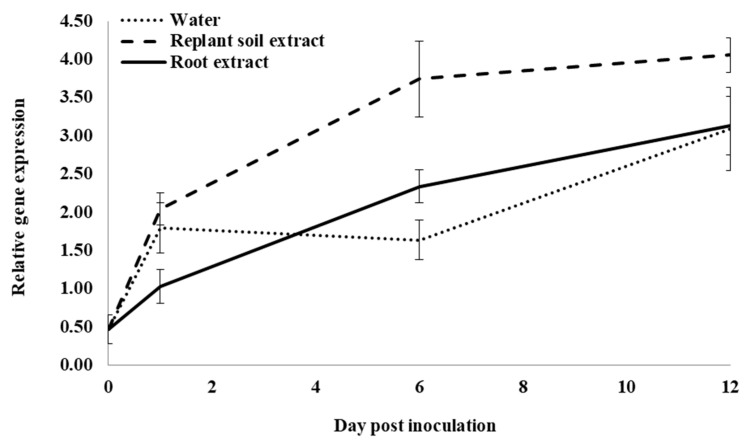
Effect of water, root extract or previous ginseng soil extract on expression of the *P. quinquefolius* acidic β-1-3-glucanase gene (*PqGlu-1*) in roots following infection by *I. mors-panacis*. Relative RT-PCR of *PqGlu-1* expression in roots of *P. quinquefolius* treated with water (**······**), 100 mg/mL ginseng root extract (**⸺⸺**) or 100 mg/mL previous ginseng soil extract (**- - - - **), followed 2 h later by inoculation of *I. mors-panacis* isolate IMP.ND4Z15 on the wounded roots. Semiquantitative RT-PCR was performed with co-amplification of *PqGlu-1* and a constitutive control, *PqIF3G1*. Each data point represents the mean of three replications, with error bars showing standard deviations.

Following infection of control water-treated roots, expression of an acidic glucanase (*PqGlu-1*) significantly increased from 0 to 1 dpi, but it did not significantly change from 1 to 6 dpi. It then significantly increased from 6 to 12 dpi (Figure 7). At 1 dpi, expression was not significantly different in roots treated with water or previous ginseng soil extract; however, both were significantly higher than roots treated with root extract by 1.7- and 2-fold, respectively. At 6 dpi, expression was significantly higher in roots treated with previous ginseng soil extract by 2.3- and 1.6-fold compared to roots treated with water or root extract, respectively. Root extract treatment resulted in significantly higher expression than water by 3-fold. At 12 dpi, expression in roots was significantly higher with previous ginseng soil extract compared to water and root extract treatments, which were not significantly different from each other. Like all the other genes examined, except *PqCPI*, *PqGlu-1* expression was induced by infection in control roots, but this was the only gene where treatment with previous ginseng soil extract resulted in a greater increase rather than a suppression of gene expression following infection.

## 3. Discussion

Larger lesions produced by *I. mors-panacis* were observed as the concentration of previous ginseng soil extract was increased, but this did not occur with increasing concentrations of root extract or hopyard soil extract. This indicated a direct dose response from some compound(s) in the previous ginseng soil extract that caused the roots to become more susceptible to the pathogen, and that those compound(s) were absent in soil not previously used for ginseng production or ginseng root extracts.

One possibility is that compounds in previous ginseng soil were ginsenosides as they were found in previous ginseng soil but were absent in the hopyard soil. Root extract contained seven PPDs and three PPTs matching those commonly reported in the roots of *P. quinquefolius* [20]. However, previous ginseng soil extract contained approx. 0.01% of the ginsenoside concentration found in root extract, and R1, Rb1 and Rc + Rb2 detectable in roots were not detectable in soil. The lower concentrations would be related to the amounts in root exudates and their persistence in the soil. The soil in this study was used one year after ginseng had been harvested, and thus low concentrations would not be surprising. One reason for the lack of certain ginsenosides in soil found in roots could be that those ginsenosides were not secreted from the roots. However, Nicol et al. [21] reported that extracted ginsenosides in daily water washes of 2-year-old *P. quinquefolius* plants growing in pots with coarse silica sand contained all the same ginsenosides found in roots, indicating that all ginsenosides can be secreted by roots. A second possibility is that some ginsenosides secreted by roots were converted to other types in soil, such as by ginseng soil bacteria that can deglycosylate ginsenosides [22,23,24,25]. For example, a ginseng soil bacterium could convert Rb1 into F2 and GXVII [23], and a human fecal bacterium could convert Rc and Rb2 into F2 [26]. With 1-year-old post-harvest field soil, there would be a considerable period of time for ginsenosides to interact with various biological and chemical factors in the soil. However, no forms of ginsenosides were detectable one year after harvesting that were not present in the root extract.

Among the genes examined in this study, *PqChi-1*, *PqPR5*, *PqPR10-2*, *PqSPD* and *PqGlu-1* showed significant induction at 1 dpi due to *I. mors-panacis* infection in control water-treated roots, indicating that they are root rot responsive genes. Some genes showed continued elevated expression to 12 dpi, whereas others showed reduced expression by 6 to 12 dpi. *PqChi-1* encodes a type of class I basic chitinase, which can degrade the cell wall of phytopathogenic fungi [27] and are up-regulated by necrotrophic pathogens and regulated by JA and ET [28]. In *P. ginseng*, *PgChi-1* showed increased expression due to JA and infection by the necrotrophic fungus, *B. cinerea* [13]. *PqPR5* encodes a type of thaumatin-like protein, which can attack the cell wall of fungi [29] and are regulated by JA and ET [30]. In *P. ginseng*, *PgPR5* expression was induced by infection of the necrotrophs, *P. ultimum*, *B. cinerea* and *Rhizoctonia* [16]. *PqPR10-2* encodes a type of small acidic ribonuclease that can be induced by JA/ET and suppressed by SA [31]. In *P. ginseng*, *PgPR10-2* expression was induced by JA and infection of *C. gloeosporoides*, *P. capsici, A. solani* and *B. cinerea* [18]. *PqSPD* encodes a type of spermidine synthase that produces different polyamines involved in many physiological processes [32]. In *P. ginseng*, *PgSPD* expression was significantly induced by JA and abiotic factors, such as salt stress [19]. *PqGlu-1* encodes a type of acidic β-1,3-glucanase that is induced by SA and can attack the cell walls of fungi [33,34]. In *P. ginseng*, *PgGlu-1* showed increased expression when SA was applied to transformed *P. ginseng* tissue culture cells [14], but expression was not reported for diseased plants.

Triggered defenses against necrotrophic pathogens are primarily regulated by JA and ET [35]. *Ilyonectria mors-panacis* has been described as a necrotroph, producing root symptoms of a brownish-colored lesion up to the entire rot of the root cortex, known as disappearing root rot [5]. Infection of *P. ginseng* roots with *I. mors-panacis* or *I. robusta* showed that they could induce expression of both JA and SA marker genes [15]. Thus, induced expression of the JA and SA-related genes in this study would be expected. Even defense genes that are induced by JA can also be induced by SA, such as *PgPR10-2* expression that was induced by JA, reaching its highest expression level 4 h after application, while it was also induced by SA with maximum expression at 12 h [18].

The only gene that did not show induction following infection by *I. mors-panacis* of control water-treated roots was the cysteine protease inhibitor gene, *PqCPI*. Cysteine protease inhibitors are involved in processes, such as response to environmental stimuli, programmed cell death and degradation of proteins [36]. The expression of *PgCPI* in *P. ginseng* was induced by JA and infection by *B. cinerea* [17]. Thus, its lack of induced expression by *I. mors-panacis* infection was unexpected, but the gene in *P. ginseng* could be regulated differently than the highly similar gene in *P. quinquefolius*.

At 1 dpi for roots treated with root extract, expression of the genes was never significantly different than in water-treated roots, except for a lower expression of *PqCPI* at 1 dpi. However, expression at 6 or 12 dpi was higher with root extract treatment than with water treatment for *PqPR10-2*, *PqGlu-1* and *PqSPD*, indicating that there may be compounds in root extract that may later affect the host response to *I. mors-panacis* infection. In contrast, previous ginseng soil extract treatment resulted in all the genes showing less expression than in control roots at 1 dpi, except for *PqGlu-1,* which was the same as control roots. Later in the interaction at 6 and/or 12 dpi, *PqChi-1*, *PqPR5*, *PqPR10-2* and *PqSPD* continued to show supressed expression relative to the control. Thus, all the significant differences for the genes related to JA, ET or necrotrophic infection were because of lowered expression at one or more time points post infection with previous ginseng soil extract treatment, indicating that it had a general suppressive effect on JA/ET-related defense gene expression.

In contrast to JA/ET-related defense gene expression, expression of the SA-related gene, *PqGlu*, showed higher expression with previous ginseng soil extract treatment. SA and JA/ET can have antagonistic effects on plant defenses [37]. Some necrotrophic pathogens, such as *B. cinerea*, can induce the SA response to suppress JA-related defense genes in tomato by producing exopolysaccharides [38]. Thus, greater induction of *PqGlu-1* expression by previous ginseng soil extract in infected roots could have been part of SA antagonism to JA/ET-related defense genes, or vice versa. Decreased JA/ET-regulated defenses could result in greater root rot [12].

The compound(s) in soil previously planted with ginseng that are responsible for the larger lesions and reduced JA/ET-related gene expression in treated roots are unknown. The lack of any novel ginsenoside in previous ginseng soil extract, which increased lesion size, compared to ginseng root extract, which did not, does not support a role for a ginsenoside being involved. Ginsenosides with allelopathic activity to ginseng have been reported to be R1, Rg1, Re, Rb1 and Rd [39]. Those were all found in much higher amounts in the ginseng root extract than in the previous ginseng soil extract. Thus, one would have expected root extract to have suppressed defense gene expression if they were responsible. However, soil previously planted with ginseng could have a variety of compounds in it. A comparison of healthy and root rot ginseng soil revealed that nine organic acids were significantly higher in root rot soil, including benzoic acid and salicylic acid, as well as other compounds with allelopathic activity [40]. Benzoic acid and salicylic acid from ginseng roots, for example, have biological activity in ginseng, such as inhibition of the growth of the radicles and shoots of *P. ginseng* seedlings [6]. Preliminary HPLC analysis of the compounds in previous ginseng soil that are responsible for increased lesion sizes in this study indicates that they are present at extremely low levels. Compounds that affect the innate immune system of plants can be effective at very low concentrations. For example, nanomolar concentrations of chitin oligosaccharides were able to trigger immune responses in suspension-cultured plant cells [41], and Gram-negative plant pathogenic bacteria are able to secrete sufficient amounts of effectors to suppress the immune system of plants only using the type III secretion system [42].

## 4. Conclusions

This study has shown that there are compound(s) in soil previously planted with ginseng that can increase the severity of root rot of ginseng compared to roots treated with either extracts from ginseng root or soil not previously used for ginseng production. The greater root rot could be related to the suppressed JA/ET-related defence mechanisms of ginseng roots allowing *I. mors-panacis* to be more aggressive, perhaps eventually resulting in plant death due to disappearing root rot, which is often associated with ginseng replant disease [43]. If the compound(s) is conclusively found to be responsible for the greater root rot, then ginseng replant disease can be considered as an acquired or secondary immunodeficiency due to an extrinsic factor affecting the host’s immunity [44]. Thus, ginseng replant disease may be due to a plant immunosuppressor compound(s) in previous ginseng soil that allows for more severe root rot by *I. mors-panacis*.

## 5. Materials and Methods

### 5.1. Biological Materials

*Ilyonectria mors-panacis* isolate IMP.ND4Z15, originally isolated from the infected roots of *P. quinquefolius* grown in a commercial garden not previously planted with ginseng near Simcoe, Ontario, was kindly provided by Amy Fang Shi, Ontario Ginseng Growers Association, Simcoe, ON. The isolate was grown on PDA for 4 weeks in the dark at 22 °C and stored at −80 °C by suspending conidia into 10% sterile glycerol (Fisher Scientific, Mississauga, ON, Canada). Three-year-old *P. quinquefolius* roots, obtained from commercial ginseng gardens in soil not previously used for ginseng production near Simcoe, Ontario, were rinsed with tap water and stored at 4 °C. Soil was collected from a one-year post harvest ginseng field, and soil not previously used for ginseng production was collected from a hopyard at the University of Guelph, Research Station, Simcoe, ON, Canada.

### 5.2. Root and Soil Extraction

Root and soil ginsenosides were extracted as per Dai and Orsat [45]. Soil (200 g) was air-dried and then mixed with 600 mL of 80% methanol (MeOH) and shaken at 175 rpm on a gyratory shaker (New Brunswick Co., Edison, NJ, USA) overnight at room temperature. The mixture was vacuum filtered using No. 4 qualitative filter paper (Whatman, Maidstone, UK), and the methanol in the filtrate was evaporated under vacuum at 40 °C. The dry residue was weighed, and a 0.1-mg/mL solution in 80% methanol was prepared for LC-MS analysis or 0.1 mg/mL in dsH_2_O for root lesion assay. The solution was then centrifuged (Beckman Coulter, High Wycombe, UK) at 10,000 rpm for 5 min and filtered using a 0.22-μm membrane (Whatman). Methanol extraction of *P. quinquefolius* roots was performed as described above, except using washed air-dried roots that were ground in a mortar and pestle.

### 5.3. Detached Root Assay

*Panax quinquefolius* roots were surface sterilized with 75% ethanol for 10 min followed by 5% bleach for 5 min. The roots were then thoroughly washed with dsH_2_O, and holes approximately 1.5 mm wide and 9 mm deep were created on the roots with a sterilized needle. An amount of 15 µL dsH_2_O, root extract, previous ginseng soil extract or hopyard soil extract were placed into the holes. After 2 h, 15 µL of a suspension of 1 × 10^6^ conidia/mL in sdH_2_O was added into each hole, and the roots were kept in sterile Petri dishes at 22 ± 2 °C. Conidia were obtained from *I. mors-panacis* isolate IMP.ND4Z15 cultured on PDA for 4 weeks in the dark. Control roots (negative control) were prepared as above, but they were inoculated with only dsH_2_O. Roots were collected at day 0 (pre-inoculation), 1, 6 and 12 dpi (days post-inoculation) by cutting 0.3 cm from each side of the wound/lesion on the root. The samples were immediately frozen in liquid nitrogen and kept at −80 °C. Lesion areas were determined from 3 replications, with six lesions measured per replication at 12 dpi by tracing the lesion area on acetate sheets, and the areas were quantified using ImageJ software version 1.22 (https://imagej.net (accessed on 1 November 2017)). Data were compared by analysis of variance (ANOVA) using Minitab version 16, and mean comparisons were performed using Fisher’s LSD Test with a level of significance at *p* = 0.05.

### 5.4. RNA Extraction

Total RNA was extracted with 3 replicates for each time point from 0.5 g of the frozen roots by homogenizing in a chilled mortar and pestle, and by adding 1 mL One Step-RNA Reagent (BioBasic, Markham, ON, Canada) and following the protocol of the manufacturer. Briefly, the homogenate solution was centrifuged at 12,500 rpm at 4 °C for 12 min and the supernatant was mixed by chloroform finger-vortex and centrifuged at 12,000 rpm at 4 °C for 15 min. The upper clear layer of the mixture was then mixed with isopropanol, NaCl (1.2 M) in DEPC H_2_O and sodium citrate (0.8 M) in DEPC H_2_O and gently mixed at 22 °C for 15 min and then centrifuged at 12,000 rpm at 4 °C for 15 min. The pellet was collected and washed with 1 mL of 70% ethanol, allowed to air dry, and then the pellet was dissolved in 50 μL of DEPC H_2_O, and 5 μL was separated on a 0.1% agarose-TBE gel to examine if bands for the 28S and 18S subunit rRNA bands were present. The concentration of the RNA was determined using a NanoDrop^TM^ Lite Spectrophotometer (ThermoFisher, Mississauga, ON, Canada), and then adjusted to 500 ng/μL in DEPC H_2_O. RNA was converted into cDNA using a Quanta Biosciences qScript^TM^ cDNA SuperMix (Quantabio, Beverly, MA, USA), following the manufacturer’s instructions.

### 5.5. Primer Design Used in Relative RT-PCR

The genes of *P. quinquefolius* chosen for examination of gene expression were based on *P. ginseng* genes previously shown to have altered expression related to disease resistance or defense hormones. These were *PqChi-1* based on a basic chitinase 1 gene (FJ790420) that had a 90-fold increase in expression in *P. ginseng* infected by *B. cinerea* [13]; *PqPR5* based on a vacuolar neutral PR5 gene (GQ452234) that had 2.5-, 4-, 4.5- and 4-fold increases in expression in *P. ginseng* infected by *B. cinerea*, *Colletotrichum gloeosporioides*, *Pythium ultimum*, or *Rhizoctonia solani*, respectively [16]; *PqCPI* based on a cysteine protease inhibitor gene (GU001147) that had a 73-fold increase in expression in *P. ginseng* infected by *B. cinerea* [17]; *PqPR10-2* based on a PR10-2 (GU086324) that had 17-, 23- and 10-fold increases in expression in *P. ginseng* infected by *C. gloeosporioides*, *Phytophthora capsici*, or *Alternaria solani*, respectively (Pulla et al., 2010); *PqGlu-1* based on an acidic β-1,3-glucanase gene (DQ015705) that had a 10-fold increase in expression in rolC-transformed *P. ginseng* cells by SA [14] and *PqSPD* based on a spermidine synthase gene (GQ229380) that had 41.5-, 39-, 20-, 6- and 15-fold increases in expression in *P. ginseng* by salt stress, chilling, ABA, mannitol and JA, respectively [19]. The constitutive control gene was *PqIF3G1* based on the eukaryotic translation initiation factor, IF3G1 gene of *P. ginseng* (KU215663.1) that had been shown to be one of the most stable constitutive control gene in real-time quantitative RT-PCR among several constitutive candidate genes in 5-year-old *P. ginseng* across different tissues (roots, stems, leaves and flowers and embryogenic calli) [46]. The nt sequences of the *P. ginseng* genes of interest and constitutive gene described above were obtained from the NCBI GenBank NR database (http://www.ncbi.nlm.nih.gov/ (accessed on 2 July 2017)).

A BLASTN search of the GenBank NR database was performed for each gene to collect the two to five most closely matching nt sequences, and the sequences were aligned using MUSCLE (https://www.ebi.ac.uk/Tools/msa/muscle/ (accessed on 3 July 2017)). A non-target sequence for each gene was chosen by selecting the closest matches to the query gene. Primer sequences were chosen based on 100% nt match to the target gene and 71–98% nt match to the non-target gene among the sequences of each alignment. The Oligo Analyzer program (https://www.idtdna.com (accessed on 3 July 2017)) was used to test for any possible self-dimers, hairpin loops or hetero-dimers as well as to determine the GC content and melting temperature. To check the specificity of the designed primers, a BLAST search of the primer sequences was performed against the GenBank NR database, and the primers that had 100% nt identity with the sequence of the gene of interest and 90–95% identity to non-target sequences were selected (Table 2).

### 5.6. Semiquantitiive RT-PCR

RT-PCR reactions consisted of 0.4 µL of cDNA, 0.35 µL 5 µ/µL *Tsg* polymerase (Bio Basic, Markham, Canada), 10× *Tsg* buffer, 10 mM Mg^2+^, 20 mM dNTPs, 25 µM forward and reverse primers for the gene of interest and 25 µM forward and reverse primers for *PqIF3G1*, in a total volume of 15 µL [47]. Amplification conditions were 1 cycle at 94 °C for 3 min followed by 27 cycles of 94 °C for 30 s, 55 °C for 1 min and 72 °C for 1 min, and finally 1 cycle of 72 °C for 10 min. An Eppendorf AG 22331 Thermocycler (Eppendorf, Hamburg, Germany) was used to carry out the reactions. The PCR products were then run on 1.5% TAE agarose gel containing 0.006% ethidium bromide, and the bands were visualized using an UV transilluminator and imaged using a CCTV camera. The number of pixels in the band for the gene of interest and *PqIF3G1* in each lane was quantified from a PDF file of the gel image using ImageJ software version 1.22 (https://imagej.nih.gov/ij/ (accessed on 3 March 2018)), and the ratio of the number of pixels of the gene of interest and the constitutive gene were calculated to determine relative gene expression. The assay was repeated 3 times for each gene.

### 5.7. HPLC-MS Analysis

High-performance liquid chromatography (HPLC) was used to determine the ginsenosides in the extracts, as per Ivanov et al. [48], with 3 replicates. Dried extracts (0.1 g) were dissolved in 1 mL 80% methanol and then filtered using a 0.22-μm membrane (Whatman). The filtered centrifuged extracts (10 μL) were injected onto a ZORBOX Eclipse Plus C8 column (2.1 × 50 mm, 1 μm, Agilent Technologies, Santa Clara, CA, USA), and eluted with a gradient of solvent B (90% acetonitrile containing 0.1% formic acid and 1 mg/L sodium acetate) and solvent A (0.1% formic acid and 1 mg/L sodium acetate) starting with 25% solvent B/75% solvent A, held for 1 min, followed by a linear gradient to 35% solvent B over 2 min, then 95% solvent B over 6 min, and maintained at 95% solvent B for 1 min before returning to 25% solvent B/75% solvent A. The flow rate was 0.4 mL/min, and the eluent was monitored at 203 nm before infusion into an Agilent 6320 TOF mass spectrometer through a dual spray electrospray ionization (ESI) source with a gas temperature of 325 °C flowing at 12 L/min, and a nebulizer pressure of 45 psi. The fragmentor voltage was 120 V with a Vcap of 4500 V. Automated internal calibration was performed using reference ions 121.0508 and 922.0096. The column was conditioned at 25% solvent B/75% solvent A for 9 min between samples and maintained at 40 °C. Ginsenosides were detected as their Na^+^ adducts in positive ion mode (M + Na^+^H).

## Figures and Tables

**Table 1:** Ginsenosides detected in *P. quinquefolius* root, previous ginseng soil and hopyard soil. The ginsenosides were extracted using 80% methanol and analyzed using HPLC with mass spectrometery (MS) on positive mode. Means of each ginsenoside followed by the same upper-case letter are not significantly different according to Fisher’s LSD at *p* = 0.05. Each mean is from three replications.

Ginsenoside	Type	Root (AU ^a^)	Previous Ginseng Soil (AU)	Hopyard Soil (AU)
R1	PDD ^b^	6.62 × 10^6^	ND ^d^	ND
Rb1	PDD	1.55 × 10^7^	ND	ND
Rc + Rb2	PDD	3.48 × 10^6^	ND	ND
Rd	PDD	8.16 × 10^6^ A	2.69 × 10^4^ B	ND
GXVII	PDD	4.55 × 10^6^ A	1.78 × 10^4^ A	ND
F2	PDD	2.04 × 10^6^ A	5.04 × 10^3^ B	ND
Total	PDD	4.04 × 10^7^ A	4.97 × 10^4^ B	ND
Rg1	PTT ^c^	1.11 × 10^7^ A	3.28 × 10^3^ B	ND
Re	PTT	4.75 × 10^7^ A	1.18 × 10^4^ B	ND
Rf	PTT	3.28 × 10^7^ A	5.08 × 10^4^ B	ND
Total	PTT	9.14 × 10^7^ A	6.59 × 10^4^ B	ND
Total	PDD + PTT	1.32 × 10^8^ A	1.16 × 10^5^ B	ND

^a^ AU = arbitrary unit. ^b^ PPD = protopanaxadiol. ^c^ PPT = protopanaxatriol. ^d^ ND = not detected.

**Table 2:** Primers designed for the amplification of *P. quinquefolius* genes in this study.

Target Gene	Primer Name	Forward (F) and Reverse (R) Primer Sequences, 5′-3′	Product Size
*PqCHI-1*	PQChi-F1 PQChi-R1	CACTAATTGCCAAAGCCAGTG GGGTTGTTTATTAGGTCCACTCC	493 bp
*PqGLU*	PQgluc-F1 PQgluc-R1	TCCTCCATCACTTGGTTCCTT TCATCAAACATAGCAAACAGATAAGT	438 bp
*PqCPI*	PQCPI-F1 PQCPI-R2	TCAGAACAGTGCCGAGATTG AGTTGGACCTCTGTTGGATGG	383 bp
*PqSPD*	PQSPD-F1 PQSPD-R1	GCCGGGAGAAGCACACTC GCTCTTGTGCTGGACCTATGG	470 bp
*PqPR10-2*	PQPR10-2-F1 PQPR10-2-R1	TCCAAAAGACCGAAACCCAGG GATGCCACCGATAAGTCAATCC	417 bp
*PqPR5*	PQPR5-F1 PQPR5-R1	TAGCCGAATACGCCCTAAACC GGGCAAGTAAATGTGCTGGTT	329 bp
*PqIF3G1*	PQIF3G1-F1 PQIF3G1-R1	GGTGCTGTTCTCATGGTATGC GGGTATGACAATTTAATCCTTCGTGT	451 bp
*PqIF3G1*	PQIF3G1-F2 PQIF3G1-R2	CCAAGCATGAGAGCAGGTG AAGGAAGATGCAGAGAGAGCC	237 bp

## Data Availability

All data, tables and figures in this manuscript are original.
